# Dynamics of bacterial growth, and life-history tradeoffs, explain differences in soil carbon cycling due to land-use

**DOI:** 10.1093/ismeco/ycaf014

**Published:** 2025-01-30

**Authors:** Cassandra J Wattenburger, Evangeline Wang, Daniel H Buckley

**Affiliations:** School of Integrative Plant Science, Bradfield Hall, Cornell University, Ithaca, NY 14853, United States; Department of Microbiology, Cornell University, Ithaca, NY 14853, United States; School of Integrative Plant Science, Bradfield Hall, Cornell University, Ithaca, NY 14853, United States; Department of Microbiology, Cornell University, Ithaca, NY 14853, United States

**Keywords:** soil, carbon cycling, life history, growth dynamics, mortality, microbial loop, necromass, microbial carbon pump

## Abstract

Soil contains a considerable fraction of Earth’s organic carbon. Bacterial growth and mortality drive the microbial carbon pump, influencing carbon use efficiency and necromass production, key determinants for organic carbon persistence in soils. However, bacterial growth dynamics in soil are poorly characterized. We used an internal standard approach to normalize 16S ribosomal RNA gene sequencing data allowing us to quantify growth dynamics for 30 days following plant litter input to soil. We show that clustering taxa into three groups optimized variation of bacterial growth parameters in situ. These three clusters differed significantly with respect to their lag time, growth rate, growth duration, and change in abundance due to growth (ΔN_g_) and mortality (ΔN_d_), matching predictions of Grime’s CSR life-history framework. In addition, we show a striking relationship between ΔN_g_ and ΔN_d_, which reveals that growth in soil is tightly coupled to death. This result suggests a fitness paradox whereby some bacteria can optimize fitness in soil by minimizing mortality rather than maximizing growth. We hypothesized that land-use constrains microbial growth dynamics by favoring different life-history strategies and that these constraints control carbon mineralization. We show that life-history groups vary in prevalence with respect to land-use, and that bacterial growth dynamics correlated with carbon mineralization rate and net growth efficiency. Meadow soil supported more bacterial growth, greater mortality, and higher growth efficiency than agricultural soils, pointing toward more efficient conversion of plant litter into microbial necromass, which should promote long-term C stabilization.

## Introduction

Soil microbial communities are critical to the formation and persistence of soil organic carbon (SOC). SOC represents 70% of terrestrial carbon stocks worldwide, and SOC dynamics are a major driver of the global C-cycle [1]. Plant residues are transformed by microbial metabolism and either assimilated into microbial biomass, released as microbial products (e.g.*,* wastes, exudates, and necromass), or modified by extracellular processes. Persistent SOC is formed when microbial products are stabilized by the soil matrix. SOC is continually cycled within the soil microbial community as cells grow and die, and the products of one microbe become the substrates for another. This cycling process forms the microbial C pump whose primary outputs are microbial biomass, CO_2_, and persistent SOC [2–4].

Microbial parameters, such as carbon use efficiency (CUE), are a key determinant of terrestrial C storage globally [5–7]. CUE is calculated as the mass of C retained in soil microbial biomass after some period of time. CUE is often confused with “growth yield”, which is the mass of cellular C generated per mass C consumed in bacterial cultures [8]. Growth yield is calculated for cells growing at equilibrium during exponential growth phase and is controlled entirely by the energetics and efficiency of cellular metabolism during balanced growth at equilibrium. While growth yield varies between species and growth conditions, it is invariant with respect to time. In contrast, CUE is integrated across all cells in a community; it is sensitive to the yield of cells but is also influenced by variation in growth phase (e.g., lag phase, exponential phase, and stationary phase), mortality, and transfer efficiency within the community over time. Living microbial biomass itself represents only a small portion of SOC, with cellular byproducts and necromass comprising up to 80% of SOC by mass [5, 9–12]. Microbial necromass has molecular properties that favor organo-mineral stabilization and entombment, which promote SOC persistence [5, 10, 11, 13, 14]. Therefore, to comprehend the mechanisms that control CUE and promote SOC storage, we must characterize the growth and death dynamics that drive the microbial C pump in soil.

Life history frameworks provide a paradigm for predicting growth dynamics on the basis of physiological or evolutionary tradeoffs that constrain fitness. For example, the copiotroph-oligotroph framework, which is described by Monod kinetics [15], posits a physiological tradeoff between growth rate and substrate affinity, in which “copiotrophic” strategists are adapted for fast growth in high-resource conditions, and “oligotrophic” strategists are adapted to optimize substrate uptake in resource-limited conditions [16–18]. This framework predicts that copiotrophic taxa will dominate nutrient rich habitats, while oligotrophs dominate nutrient poor habitats, and that microbial processes in such habitats are defined by the physiological traits of these two microbial groups. However, Monod kinetics describe competitive interactions when substrate concentrations are at equilibrium, such as occurs in a chemostat, an assumption never met in soil, and the copiotroph–oligotroph framework has been criticized for its simplicity and inability to capture the complexity of microbial growth dynamics as they occur in soil [19].

The CSR life history framework, conceived originally for plants, posits that environmental variation in resource availability and disturbance frequency impose ecological tradeoffs on organismal energy allocation to growth, resource acquisition, and survival [20]. In the CSR framework, as adapted for microorganisms [19, 21, 22], ruderal (R) taxa optimize fitness by maximizing growth rates and are favored when resources are highly variable over time. Fast growth allows R strategists to exploit transient resource pulses and periods of disturbance when resource availability exceeds the metabolic demand of the community and competition is not constrained by Monod kinetics. Competitive (C) taxa optimize fitness by maximizing resource acquisition and are favored when resources are high and temporal variability (or disturbance) is low. C strategists seek to acquire resources by investing energy in extracellular products (e.g., secreted enzymes, siderophores, and antibiotics) and structures (e.g., biofilms and hyphae) which facilitate nutrient acquisition, but which are highly sensitive to disturbance. Stress tolerant (S) taxa optimize fitness by maximizing tolerance to resource limitation (i.e., scarcity). S strategists seek to minimize metabolic costs to remain metabolically active at low resource availability.

The CSR framework differs from the copiotroph–oligotroph framework in several ways. First, CSR adds an additional tradeoff that helps it to explain greater variability than the copiotroph–oligotroph framework. Second, CSR does not require the assumption of equilibrium growth and is predicated upon the idea that environmental conditions change over time. Finally, CSR posits “ecological tradeoffs”, while the copiotroph–oligotroph framework posits “physiological tradeoffs”. Cellular physiology is an important determinant of fitness, but the fitness of populations is also influenced by numerous ecological determinants (e.g., antagonism, facilitation, predation, and density dependent effects, among others), which can produce complex tradeoffs not predicted from Monod kinetics. Various flavors of the CSR framework have been proposed for microorganisms [19, 21, 23, 24], and there is growing evidence that CSR life-history models are useful for understanding microbial impacts on ecosystem function [22, 25–27].

To define bacterial life history strategies and describe the operation of the microbial C pump in soils, we need to observe non-equilibrium growth dynamics as they occur in soil. Bacterial growth rates in situ have been shown to correlate strongly with C mineralization [28], suggesting that in situ growth measurements will be useful in modeling microbial contributions to the soil C-cycle. It is clear that CUE is highly dependent on environmental and ecological context [29–31], being driven by microbial growth efficiency as well as rates of death and necromass turnover. Soil C models predicated on parameters derived from Monod kinetics ignore the ecological interactions that define fitness in soil. Finally, growth rates tend to be far slower in soil than in culture [32, 33], and bacteria in soil exhibit enormous variability in growth rates indicating tremendous diversity in physiology, beyond what we could observe in any given culture condition [33–37]. These observations all point to the importance of measuring bacterial growth dynamics as they occur in soil.

We investigated whether in situ microbial growth dynamics vary with respect to land-use and if these differences can explain variation in soil C cycling. Specifically, we measured bacterial growth and C dynamics in soils from a cultivated agricultural field and an adjacent old-field meadow. Edaphic changes driven by land-use history have major impacts on microbial community structure and function, particularly in relation to soil C cycling [33]. We hypothesized that land-use imposes constraints on microbial growth dynamics, by favoring microbes with different life history strategies, and that differences in growth dynamics control variation in CO_2_ mineralization. We added ^13^C-labeled plant litter to soil microcosms, estimated bacterial growth and death in soils using 16S ribosomal RNA amplicon sequencing paired with the amplicon read normalization by internal standard ratio method [38, 39], and related net bacterial growth to CO_2_ mineralization. K-means clustering of bacterial taxa on the basis of in situ growth parameters, produced groups whose growth dynamics were largely consistent with predictions of the CSR model. We observed that these different life history groups varied in prevalence with respect to land-use, and we show that bacterial growth dynamics correlate with CO_2_ mineralization and growth efficiency in soils.

## Materials and methods

### Experimental design

Soil was collected from the Cornell University Agricultural Experiment Station Ketola farm site from a cultivated agricultural field (42°28′09.8N 76°25′56.2″W) and an adjacent old-field meadow (42°28′09.5″N 76°25′54.0″W) on 9 November 2021. Both fields consisted primarily of Langford channery silt loam, Erie-Chippewa channery silt loam, and Rhinebeck silt loam soil types. The agricultural and meadow soils had acidic pH (pH 5.0 and 5.5, respectively) and high soil organic matter (SOM; 5% and 8%, respectively) [40]. The agricultural field had been used historically for grain production with annual tillage and standard fertilization practices for the region. The old-field meadow had not been cultivated since 1938. From each plot, 60, 2.5 cm diameter, 10 cm depth cores were taken along a diagonal transect and homogenized. Soil was dried, sieved to 4 mm, and stored at −20°C until use.

Microcosms were prepared by adding 5 g dry weight soil to 100 mL serum bottles. Soil microcosms were capped with a foam stopper and pre-incubated for one week, in the dark, at room temperature. Following pre-incubation, each microcosm received 20 mg of either unlabeled or ^13^C labeled common bent root, root litter (*Agrostis capillaris,* >97% atom 13C, IsoLife, Wageningen, The Netherlands), which had been ground (< 1 mm particle size) and sterilized. Bent root is a grass, which was chosen because of its availability and the fact that meadow communities were dominated by functionally similar grasses. We choose to use root litter, because C inputs from decaying roots represent a major resource input to soil microbes, particularly in agricultural soils where aboveground residues are harvested. The use of ^13^C-litter allows us to track litter mineralization into ^13^CO_2_. After the litter was added, microcosms were homogenized by mixing with a sterile applicator stick, brought to 50% water holding capacity, sealed with butyl septa, and incubated for 30 days. Microcosm headspace was flushed daily (by evacuation and flushing with filter-sterilized ambient air (0.22 um)) for the first 15 days and then every second day through day 29. Microcosm headspace ^12^CO_2_ and ^13^CO2 were measured using a Shimadzu GCMS-QP2010S with a Carboxen 1010 PLOT column (Supelco, Bellefonte, PA, USA). On day 15 a hardware failure caused most headspace samples to be lost for that day, so the missing data was imputed based on the average of the neighboring time point measurements. Microcosms were sampled destructively every 12 hours from day 0–7, daily from day 8–13, every 2 days from day 15–27, and then again on day 30. Three replicates were made for each *treatment x isotope x time* condition resulting in a total of 190 microcosms to allow for destructive sampling. Soil samples were frozen immediately at −20°C following destructive sampling. Deoxyribonucleic acid (DNA) was extracted from 500 mg soil using the DNeasy PowerLyzer PowerSoil kit (Qiagen) and resuspended in 50 ml elution buffer. After DNA extraction, 1.625 pg of internal standard (modified *Aquifex aolicus* 16S rRNA gene V4 sequence, as described previously, Wattenburger & Buckley, 2023 [33]) was added to 10 uL of each DNA sample.

### DNA sequencing

A 16S rRNA gene V4 amplicon sequence library was prepared via the following protocol. Each DNA sample was amplified in duplicate using Kozich *et al.*’s 16S rRNA gene V4 region 515f–816r primers [41]. Reaction conditions were as follows: 12.5 μL of Q5 High Fidelity PCR Master Mix (New England Biolabs, Ipswich, MA USA), 0.6 μL of 4× Quant-iT PicoGreen dsDNA reagent (Invitrogen, Waltham, MA USA), 2.5 μL each of 10 μM primers, 5 ng of DNA template with internal standard, and molecular grade water to a total reaction volume of 25 μL. The thermocycler conditions were as follows: 98°C for 2 min, 30 cycles of 95°C, 55°C, then 72°C for 30 seconds each, a melt curve from 70°C–95°C with 0.5°C steps, 72°C for 5 min, and a 4°C hold. Each PCR plate included a positive control and a no-template negative control. Duplicate amplifications were pooled and normalized to equal concentrations using SequalPrep Normalization Plate Kits (Invitrogen, Waltham, MA USA). These samples were combined into a single library, gel selected at 400–500 bp using the Wizard SV Gel and PCR Clean-Up System (Promega, Madison, WI USA), and submitted for sequencing at the Cornell Institute of Biotechcnology BRC on an Illumina MiSeq instrument using V2 chemistry and 2 x 250 bp reads.

This project’s Github repository (https://github.com/cwatt/NIFA) contains all the scripts used for raw data processing and data analysis. QIIME2 v2021.4 was used to process the raw sequence data [42]. First, short sequences of less than 100 bp were discarded. Cutadapt was used to trim primer from the remaining sequences [43]. Sequences were then demultiplexed and trimmed to exclude low quality base calls and ensure equal sequence lengths for amplicon sequence variants (ASV) creation. DADA2 [44] was then used to filter, denoise, and merge the sequences into ASVs. These ASVs were classified taxonomically using the Silva v138 database [45], and a phylogenetic tree was generated by aligning the sequences with MAFFT 7 [46] and constructing it with FastTree 2 [47]. R version 3.6.3 [48] was used for all subsequent analyses unless otherwise stated. ASV count data was filtered to exclude non-prokaryotic, chloroplast, and mitochondrial sequences. Samples were rarefied to 8770 counts each using the phyloseq package [49]. Lastly, ASV counts were normalized to the internal standard count in each sample to create a normalized abundance measurement [38, 39].

### Data analysis

Growth and death were estimated using the algorithm previously published [33]. Briefly, to estimate growth or death in each time series, normalized abundance data was natural log-transformed and fit progressively to linear models using windows of three or more consecutive time points until all possible windows of time were fitted. Regression slopes that were positive (or negative for death estimates) and that passed a *P*-value threshold of less than 0.05 were recorded. A single best fit estimate was then chosen among these estimates for each time series based on the fit with the lowest slope *P*-value. Lastly, the false discovery rate was limited to 5% using a *P*-value threshold determined by simulating random time series and applying the same algorithm. From these final estimates, the specific growth or death rate (day^−1^), change in normalized abundance due to growth or death (ΔN_g_, ΔN_d_), and start and end days of growth or death were calculated (see Fig. S1 for example). To account for multiple copies, *rrn* copy numbers were predicted using PAPRICA v0.6 [50] and used to correct normalized abundance values.

Additionally, we leveraged data from a multi-substrate (amino acids, palmitic acid, cellulose, lignin, and xylose) stable isotope probing experiment conducted previously on these same soils [40]. We used nucleotide BLAST [51] to match ASVs from this study to OTUs from the previous study that incorporated ^13^C. Only matches with at least 97% sequence identity were retained. The match with the lowest *e*-value was chosen in cases where multiple OTUs matched to a single ASV with at least 97% identity. In cases where one or more OTUs matched equally well to a single ASV, the union of the substrate incorporation profiles of the two matched OTUs was used.

Growth metrics and substrate labeling profiles were used as features in k-means clustering using the R package *cluster* [52, 53] to identify groups on the basis of similarity in growth parameters and C assimilation dynamics. The averaged generation time, lag time, end of growth day, duration of growth, starting normalized abundance, ∆N_g_, and number of substrates incorporated were natural log transformed, scaled, and used to perform k-means clustering with *n* = 3 clusters. Each soil was clustered separately to avoid biases caused by the environment. Three clusters were selected for two reasons; first, principal component analysis prior to clustering showed that three components explained 80% of the cumulative variance in the data, and second, there was a clear inflection at three clusters (Fig. S2) when assessing the total within-cluster squared distance at various cluster numbers, indicating that the addition of extra clusters explains limited additional variation beyond the third cluster. In addition, most life history frameworks identify three main strategies relevant to microbial C cycling, giving a theoretical justification for three clusters [20, 22, 23, 54]. Net growth efficiency was defined as the sum of the imputed ∆N_g_ of all growing taxa during a given time interval divided by the total mass of mineralized CO_2_.

Differences between clusters were determined using nested linear models or Welch analysis of variance (ANOVA) as befitting the characteristics of the data. Contrasts were conducted with either linear models or Welch *t*-tests with Holm *P*-adjustments. Log transformations were applied as necessary to meet model assumptions. Correlations between variables were determined using Pearson correlation tests. Differences in community composition were determined using permutational analysis of variance (permANOVA). Fisher exact tests with Holm *P*-adjustments were used to test independence in the relationships between cluster membership and substrate incorporation number or preference.

## Results

### Community diversity and growth parameter estimation

Bacterial growth dynamics were assessed in microcosms over a period of 30 days by performing 16S rRNA sequencing combined with the use of an internal standard to generate normalized data suitable for analysis of growth dynamics (see Methods). Sequencing produced 50 596 raw reads per sample, with a range of 8770 to 124 583 reads (*n* = 190 samples in total). The internal standard comprised 5 ± 2.11% (average ± SD) of read counts. Only ASVs present at high frequency across microcosms are useful for growth parameter estimation, which necessitates removal of uninformative ASVs. After quality filtering, independent filtering to remove uninformative or sparse taxa, and rarefication (to 8770 sequences per sample), the dataset contained 2797 informative ASVs. The meadow soil microcosms had more informative ASVs than the agricultural soil microcosms (580 ± 84 SD ASVs, 396 ± 84 SD; Kruskal–Wallis: *P* < .0001; Fig. S3A). Community composition, as assessed by weighted-unifrac distances, differed with respect to land-use and time, and these differences were significant (permANOVA: Soil R^2^ = 0.50, *P* = .001, Day R^2^ = 0.10, *P* = .001, interaction R^2^ = 0.01, *P* = .003; Fig. S3B).

**Figure 1:**
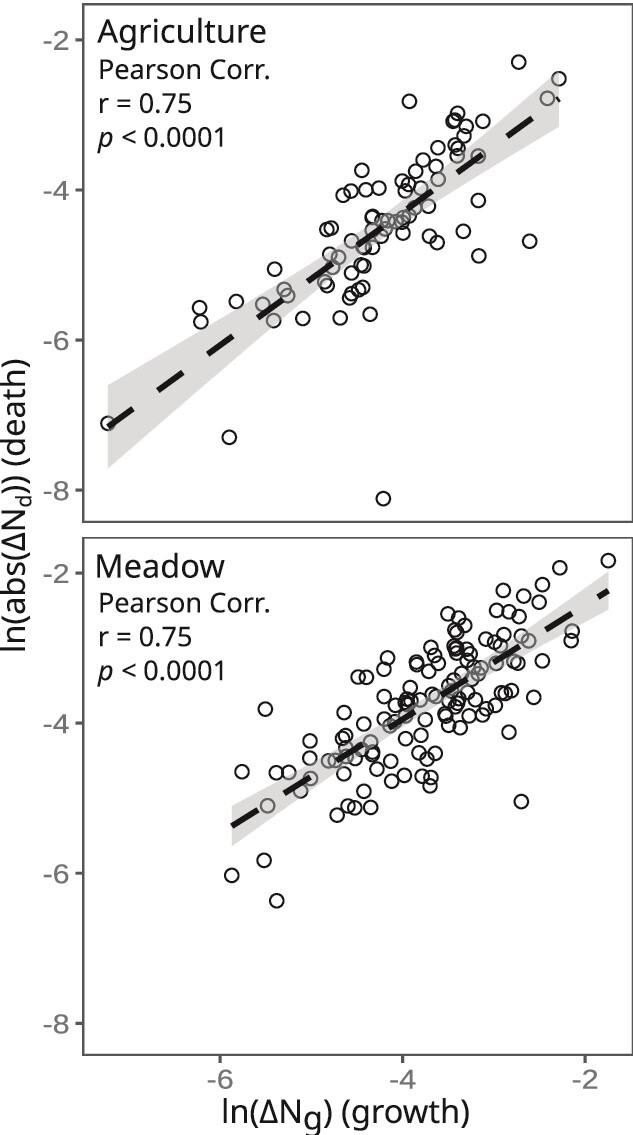
The change in bacterial abundance due to growth (∆N_g_) is tightly correlated with change in abundance due to death (∆N_d_) in agricultural (top) and meadow (bottom) soils amended with plant litter. Each point represents an ASV that had growth and/or death estimate during the 30-day incubation. Growth and death parameters for each ASV were averaged across replicates. Inset statistics show Pearson correlations with multiple test corrections. Dashed lines show linear model fit with 95% confidence intervals.

Growth estimates were obtained for 328 and 256 ASVs, and mortality estimates for 273 and 133 ASVs, in the meadow and agricultural soils, respectively. Growth and death parameters can only be estimated for ASVs that are detected consistently across time and exhibit substantial temporal autocorrelation. Growth parameter estimation is unsuccessful for ASVs that exhibit sparse and noisy patterns of abundance, which is generally seen for ASVs present at very low abundance. The phyla comprising the largest proportion of growth estimates in the meadow and agricultural soils were *Actinomycetota* (78 and 63 ASVs), *Psuedomonadota* (formerly *Proteobacteria*, 77 and 61 ASVs), and *Acidobacteriota* (47 and 28 ASVs; Fig. S4). We found no evidence that growth rates were conserved phylogenetically (Mantel correlation non-significant at all phylogenetic distances tested, data not shown). The average, standard deviation, and range (minimum, maximum) for growth and death parameters measured in each soil are summarized in Table S1. Examples of ASVs exhibiting growth or death are provided in Fig. S1. Notably, we observed a strong relationship between ΔN_g_ and ΔN_d_ in both land-use types (Fig. 1), which shows that growth in soil is tightly coupled to death.

### Characterizing growth dynamics in terms of life-history strategy

**Figure 2:**
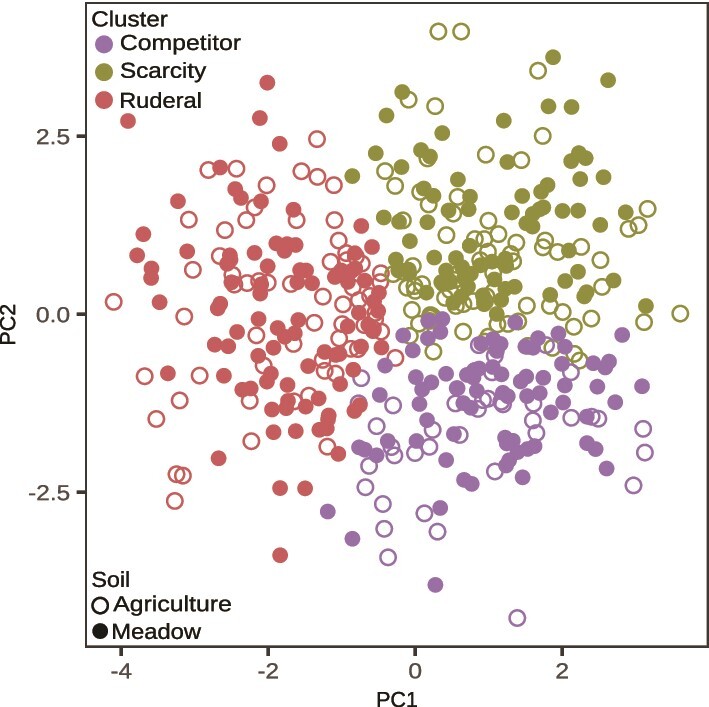
Bacterial taxa were clustered into three growth strategies on the basis of variation in their in situ growth parameters and C substrate incorporation patterns. Each point represents an individual ASV from the agricultural or meadow soil. The ordination represents principal components analysis of 7 features (average generation time, lag time, end of growth day, duration of growth, starting normalized abundance, ∆N_g_, and number of substrates incorporated) estimated for 328 and 256 ASVs observed in an agricultural and meadow soil, respectively. Clusters were determined by k-means clustering with 3 clusters selected as described in methods. Cluster names (indicated in legend) were determined subjectively as inferred from their growth parameters as determined in post-hoc analyses (see Figs 3, 4, and5).

**Figure 3:**
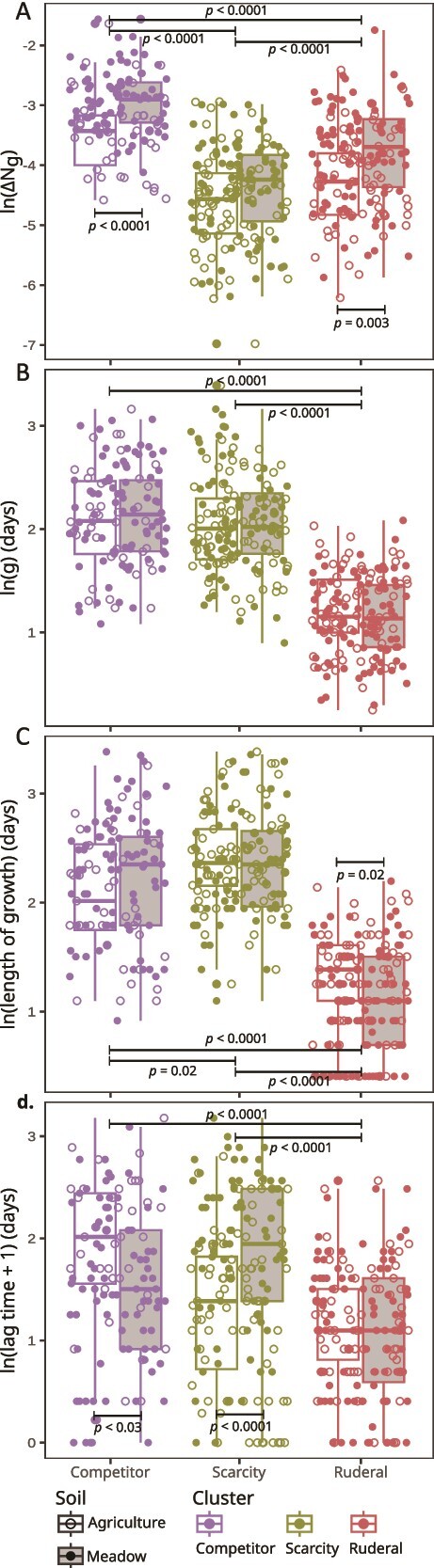
ASVs differed in ΔN_g_ (A), generation time (B), duration of growth (C), and lag time (D) based on cluster membership and soil habitat. Each point indicates the average value for an ASV from a given cluster and habitat (indicated by color and fill as defined in the legend), and boxplots (median, interquartile range, and 1.5× interquartile range) summarize these data. Cluster membership was defined as described in Fig. 2 and in methods. Horizontal bars show significant effects of cluster, and soil habitat within each cluster, based on linear models with holm *P*-adjustments. Non-significant results are not shown.

**Figure 4:**
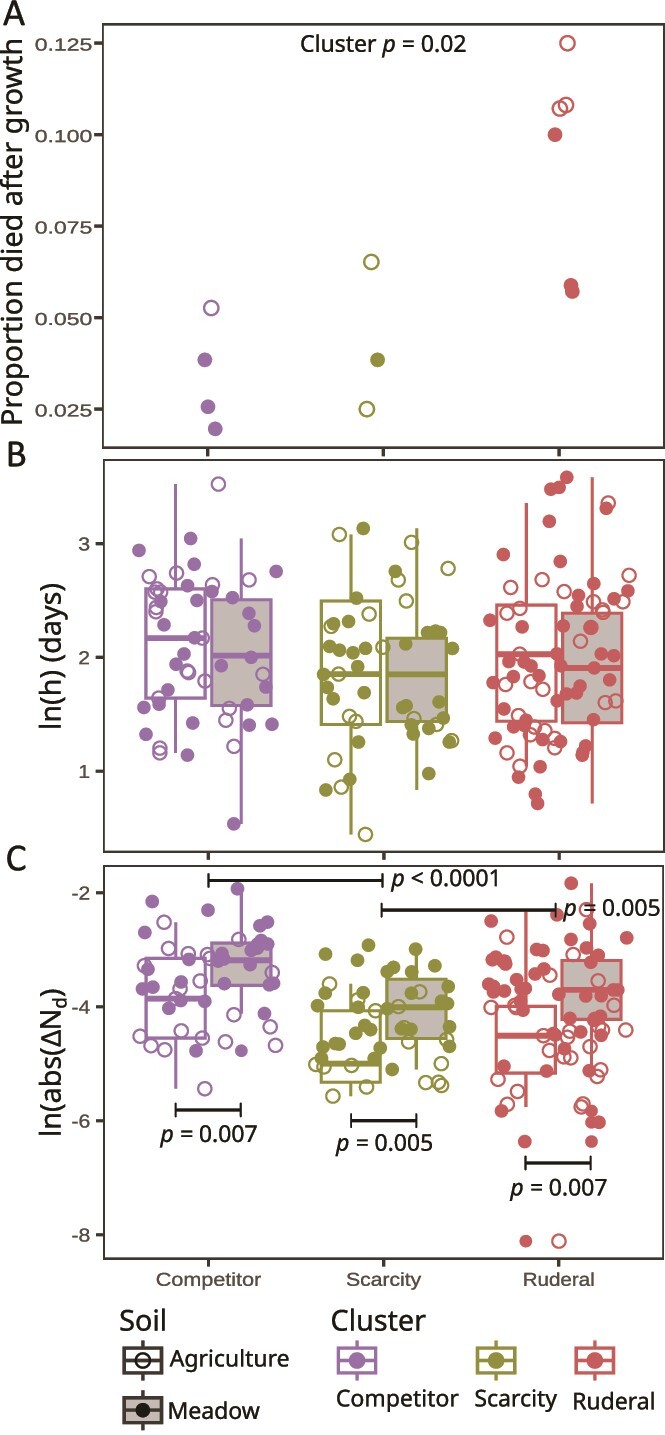
Ruderals were significantly more likely to experience mortality than competitors and scarcity strategists (A), and both ruderals and competitors experienced significantly greater mortality than scarcity adapted taxa (C). Additionally, the change in abundance due to mortality (ΔN_d_) was greater in meadow soils than in agricultural soils for all three clusters (C). The proportion of ASVs that grew and then died (A) is calculated from ASVs that produced growth and death estimates within the same replicate. Despite the observed differences in frequency and magnitude of mortality we observed no significant difference in halving times (h) between the three clusters (B). Horizontal bars indicate the test results of individual contrasts conducted using either linear models or Welch *t*-tests with Holm multiple test correction between clusters or between soils within the same cluster. Non-significant test results are not shown.

K-means clustering of ASVs was performed with respect to seven different in situ growth parameters. Principle components analysis of these growth parameters revealed that the first three principle components explained 80% of variation (Fig. S2), and so clustering was used to sort ASVs into three growth clusters (Fig. 2). We describe these three clusters as ruderal (R), competitive (C), and scarcity-adapted (S) based on how their growth parameters map to predictions of the CSR model. We use the term “scarcity-adapted” to define “S” instead of the conventional “stress tolerator” nomenclature of Grime, because plant stress as defined by Grime relates to photosynthetic energy limitation imposed by shading and low water potential. The term “stress” as applied to bacteria typically implies abiotic stress, which leads to misapplication of Grime's framework. Hence we use the term “scarcity adapted”, to clarify that S-adapted bacteria are adapted for life at low energy flux and not bacteria adapted to tolerate environmental stress (for further justification see [22]). Growth rate, ∆N_g_, lag time, growth duration, and ∆N_d_ (i.e., mortality) estimates all differed between growth clusters, and these differences were significant (nested linear models, with Holm *P*-adjustments as appropriate, *P* < .05 for each model, see Figs 3 and 4 for *P*-values of specific contrasts). Predicted R strategists had shorter lag times (Fig. 3D), faster growth rates (Fig. 3B), shorter growth duration (Fig. 3C), and a greater frequency of ASVs experiencing mortality (Fig. 4A) than C and S strategists. Predicted S strategists had the longest growth duration (Fig. 3C), while having the lowest ∆N_g_ (Fig. 3A), and the lowest ∆N_d_ (Fig. 4C). Predicted C strategists achieved the highest ∆N_g_ (Fig. 3A), though their maximal growth rate was significantly less than R and their growth duration was significantly less than S (Fig. 3C). Death rates were not observed to differ significantly between clusters (Fig. 4B).

We also show that the growth parameters of each cluster varied with respect to land-use (Fig. 3). C and R achieved greater ∆N_g_ in the resource rich meadow soils than in the agricultural soils, and these differences were significant (Fig. 3A). C strategists had significantly longer lag times in disturbed agricultural soils than in native meadow, while the opposite was true for S strategists (Fig. 3D). Meanwhile, R strategists had significantly longer growth duration in agricultural soils than in meadow soils (nested linear model: *P* < .0001, Fig. 3C). All three clusters exhibited significantly greater ∆N_d_ in meadow soils than in agricultural soils (Fig. 4C).

**Figure 5:**
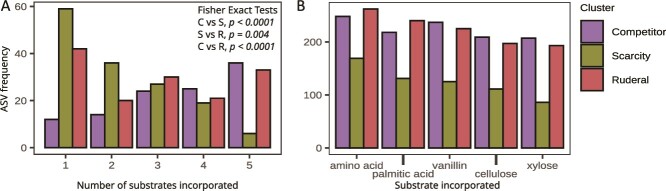
Substrate incorporation profiles for all clusters indicate that the number of substrates incorporated by an ASV depended on cluster membership (A), but there was no evidence for substrate preference (B). Inset statistics show the result of fisher exact tests for all contrasts between clusters.

We mapped ASVs to OTUs from a prior multi-substrate stable isotope probing experiment conducted in the same sites [40] to determine whether the predicted life-history clusters differ in their feeding patterns. The clusters differed in the average number of ^13^C sources they accessed (Fisher exact tests, *P* < .05 all contrasts, Fig. 5A). On average, C strategists accessed the most C sources, and S strategists accessed the fewest, while R strategists exhibited the greatest variation in substrate preferences (Fig. 5A). The three clusters did not differ in their ability to access C from individual substrates (Fig. 5B).

**Figure 6:**
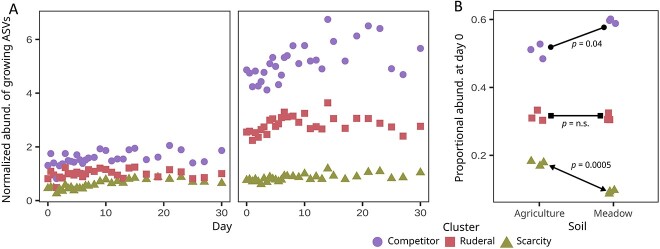
Bacterial clusters differed in their total (A) and proportional (B) abundances in agricultural (left side of panel A) and meadow (right side of panel A) soil. Each point represents the summed or proportional abundance of all ASVs within a given cluster and soil at the first time point. All clusters were more abundant in meadow soils than in agricultural soils, reflecting the higher overall abundance of DNA in these soils, but the proportion of competitors was highest in meadow soils while the proportion of scarcity strategists was highest in agricultural soils. Lines connecting data points indicate individual soil contrasts conducted using Welch *t*-tests with a Holm *P*-adjustment.

We summed the normalized abundance for all ASVs in each growth cluster to evaluate differences in cluster abundance over time with respect to land-use (Fig. 6A). All three clusters were more abundant in meadow soil than agricultural soil (Welch ANOVA: soil *P* = .02, cluster *P* = .02), which occurs because the meadow soil has significantly more DNA overall than the agricultural soil (Fig. S5). To assess native differences in community composition, we also calculated the proportional abundance of each group prior to resource addition (Fig. 6B). We show that C strategists comprise a larger proportion of the community in undisturbed meadow soils, while S strategists comprise a larger proportion of the community in agricultural soils (Welch *t*-tests with Holm *P*-adjustment: competitor *P* = .04, ruderal *P* = n.s., scarcity *P* = .0005; Fig. 6).

### Relating bacterial growth to C flux

**Figure 7:**
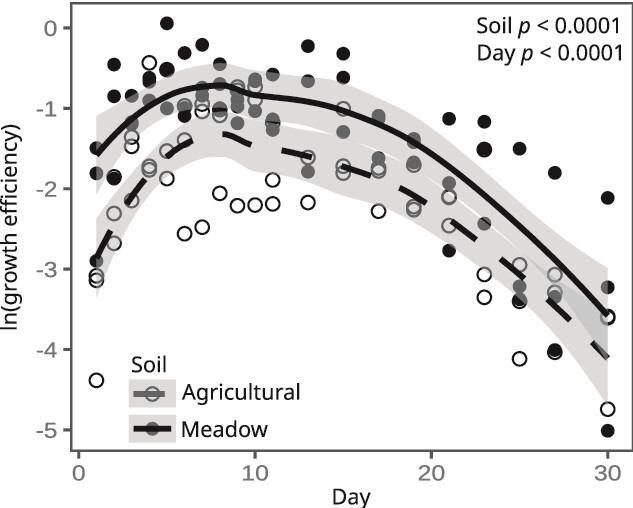
Net growth efficiency was significantly higher in meadow than in agricultural soil. Each point shows the net growth efficiency for the community for each time interval of the incubation. Lines with shaded areas depict smoothed splines and 95% confidence interval to demonstrate the temporal trend of the data for the agricultural and meadow soils.

**Figure 8:**
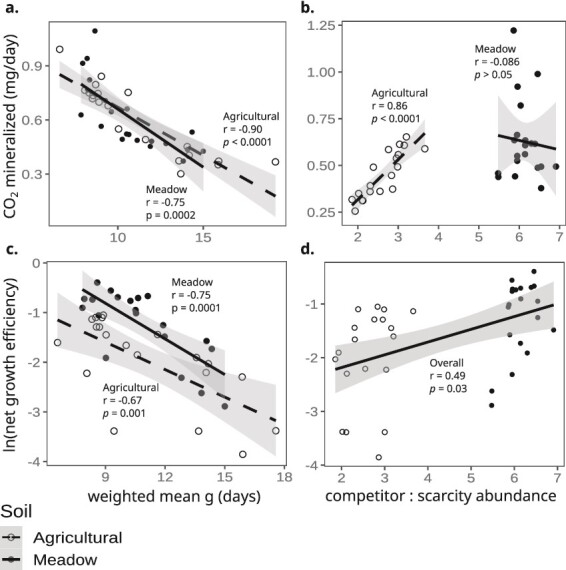
Weighted mean generation time (A and C) and cluster abundance (B and D) were correlated with CO_2_ mineralization rate (A and B) and net growth efficiency (C and D). Each point represents values averaged across replicates for a given soil and time point. Cluster representation is indicated as the ratio of competitor ASV abundance relative to scarcity adapted ASV abundance at a given time point. Inset statistics show the result of Pearson correlation tests for each soil. Dashed lines show the linear regression fit of the data while shaded areas show the 95% confidence intervals.

Total CO_2_ mineralization from either unlabeled SOM or ^13^C-labeled litter (^12^CO_2_ and ^13^CO_2_ measurements respectively) did not differ between the two soils, owing to high variability in CO_2_ flux from the agricultural soil (Welch *t*-test: *P* = n.s.; Fig. S6). However, the net growth efficiency of the community (expressed as CO_2_ generated per net gene copy per unit time) was higher in the meadow soil than in the agricultural soil (Welch ANOVA: soil *P* < .0001, day *P* < .0001; Fig. 7). CO_2_ mineralization rate and net growth efficiency were each found to strongly correlate with abundance-weighted average growth rate across time (Fig. 8A and C). Given this relationship to growth rate, we explored whether the frequency of growth clusters predicts soil C mineralization. Ruderal taxa are highly dynamic, and their abundance is likely to be noisy, hence to assess differences with respect to land-use we excluded ruderals and focused on the ratio of summed abundance for all competitive taxa (C) and scarcity adapted (S) taxa. We found that the community C:S ratio was positively correlated with CO_2_ mineralization rate in the agricultural soils, but not in meadow soils where community C:S is highest (Fig. 8B). This result suggests a tipping point in community assembly (between C:S values of 3.5 and 5.5 in Fig. 8B), below which mineralization rates are sensitive to the frequency of competitive taxa. The community C:S ratio was also weakly correlated with net growth efficiency in both agricultural (Pearson: r = 0.29, *P* = .23) and meadow soils (Pearson: r = 0.45, *P* = .04), and since these correlation coefficients did not differ significantly (Z-test: z = −0.545, *P* = .5860) we combined the data across treatments to reveal that there was a moderate positive correlation between community C:S ratio and growth efficiency across land-use (Fig. 8D).

## Discussion

Microbial growth dynamics define the mechanism of the microbial C pump as it relates to CUE and soil C storage. We show that the majority of variation in bacterial growth dynamics could be explained by sorting bacterial taxa into three growth clusters. We chose three clusters based on the large amount of variability in growth dynamics explained by these clusters, the total within-cluster squared distance, and the fact that existing life-history frameworks usually recognize three groups [17–19, 22, 23, 27, 55]. We found that each cluster contains a diverse continuum of growth parameters (Figs 2–4), consistent with prior observations [22, 24, 37]. A microorganism’s growth strategy manifests through the interaction of physiological traits with ecological context, and so a continuum of overlapping characteristics among clusters is an explicit prediction of life history frameworks [56–59]. Likewise, the CSR model predicts the existence of intermediate taxa (e.g. C-R, rather than just C and R) that have evolved differing degrees of trade-offs with respect to growth, nutrient acquisition, and survival [20].

The three clusters we obtained had growth characteristics that were consistent with Grime’s CSR framework. Taxa identified as ruderals exhibited fast growth rates, short lag times, short growth duration, and a higher likelihood of death after a period of rapid growth (Figs 3 and 4), indicative of boom-bust-like population dynamics [40], and consistent with the expectation that these taxa are adapted for rapid growth. The competitor and scarcity groups both displayed slower growth rates, longer lag times, and longer growth durations than ruderals. However, the competitors were generally capable of incorporating a wider diversity of C substrates than the scarcity strategists (Fig. 5), and they experienced larger gains in abundance (∆N_g_) during their growth period (Fig. 3A), consistent with the expectation that competitors are adapted for resource acquisition. Additionally, we observed the lowest losses in abundance due to mortality, ΔN_d,_ in the scarcity strategists (Fig. 4C), indicating that these taxa might optimize fitness by avoiding death rather than maximizing growth. Overall, these findings support the legitimacy of adapting existing ecological theory for use in microbial ecology [21, 27] and they support the application of CSR-like frameworks for describing microbial adaptive strategies.

We examined the impacts of land-use on the three bacterial life-history clusters we defined. We observed that the relative proportion of each cluster varied with respect to land-use (Fig. 6B). The total DNA yield, species richness, and total abundance of each life-history cluster were significantly lower in the agricultural soil relative to the meadow soil (Figs S3A, S5, and Fig. 6A). Ruderal strategists should have a selective advantage when disturbance frequency is high, competitors when competition for resources is high, and scarcity strategists when resources are scarce [57]. While we expected ruderals to comprise a greater proportion of the community in agricultural soils than in meadow soils, we observed a similar proportion of ruderals in both land-use types (Fig. 6B). High temporal variability in growth rate and abundance is likely a hallmark of ruderal ecology, owing to their boom-bust dynamics [22, 40], and if this is the case then their habitat abundance might be difficult to generalize from any single time point. For instance, ruderal taxa were much more abundant in the agricultural field than in the meadow when these same sites were sampled previously at a different time of the year [40]. Competitors, in contrast, had higher initial relative abundance (Fig. 6B) and shorter lag times (Fig. 3D), in meadow than agricultural soil, indicating an advantage for this growth strategy in the meadow soil. The meadow soil has more resources, less disturbance, and higher microbial abundance, which are conditions that should favor competition for resource acquisition [60–63]. Meanwhile, scarcity-adapted taxa comprised a larger proportion of taxa in the agricultural soil than in the meadow soil. Since S-strategists have significantly lower mortality than C or R (Fig. 4C), and mortality correlates strongly with growth (Fig. 1), we can hypothesize that scarcity adapted taxa achieve maximal fitness by minimizing their mortality, because low growth rates are linked to low mortality in soils. This hypothesis predicts that S-strategists exploit a fitness paradox in soil, whereby they maximize fitness by minimizing growth rate. This paradox could be driven by both physiological mechanisms (e.g. mortality could be driven by the high metabolic costs that ribosomes impose on cells when transient nutrients are depleted suddenly due to exponential growth dynamics) and ecological mechanisms (e.g., large rapidly growing populations might be more susceptible to viruses and other predators).

We found that the meadow soil had higher ∆N_g_ than agricultural soils, but similar CO_2_ mineralization rates, which indicates that the net growth efficiency of the meadow soil communities is higher than the agricultural soil communities (Fig. 7). Our measure of net growth efficiency relates CO_2_ mineralization directly to genome replication (as gene copies). This measurement differs from CUE, which is typically measured as C allocated to total microbial biomass. Fitness is determined by genetic contributions to future generations and so we argue that expressing CO_2_ mineralization directly to cell division provides a measure of efficiency that is ecologically relevant. In addition to higher ∆N_g_, we also observed that meadow soil communities had significantly higher ∆N_d_ than agricultural soil communities (Fig. 4C). This result suggests that the meadow soils produced more necromass than the agricultural soils. Efficient conversion of plant litter into bacterial biomass and ultimately into bacterial necromass may be one mechanism that allows meadow soil to sustain higher SOM levels than agricultural soils [5, 11, 12, 64].

We found that the abundance-weighted generation time was strongly correlated with both CO_2_ mineralization rate and net growth efficiency of communities over time (Fig. 8A and C). We also found a moderate correlation between the proportional abundance of competitive taxa, adapted for resource acquisition, and both CO_2_ mineralization rate and net growth efficiency (Fig. 8B). Blazewics *et al.* (2020) report a strong relationship between community growth rate and soil CO_2_ mineralization [28], suggesting that information on community growth dynamics could be useful for estimating soil C flux. However, there is disagreement about the relationship between microbial growth rate and efficiency. At the cellular level, there is evidence that growth efficiency declines as growth rate increases [65–68]. In contrast, studies that investigate the soil CUE report that growth efficiency increases with microbial growth rate [64, 69, 70], which our results also support. We propose that this discrepancy is explained by the effects of mortality, which are absent from cellular measurements of yield made in culture during balanced growth, but which have a major impact on CUE measurements made in soils. Since growth in soil is tightly coupled to death (see Fig. 1, and also [33]), we propose that mortality and recycling increase the apparent growth efficiency of soils as microbes re-use metabolic products (e.g., metabolic wastes, amino acids, nucleotides, and vitamins). Geyer *et al.* (2016) make the salient point that measurements of CUE made at the organismal, community, and ecosystem levels are all measuring different processes, resulting in inconsistent and seemingly contradictory findings [71]. More research to disentangle these confounding factors would be invaluable to understand the relationships between microbial growth dynamics and soil CUE.

In conclusion, we found that clustering taxa into three groups optimized variation of bacterial growth parameters in situ, these three groups also exhibited characteristics predicted by the CSR life history framework. These three groups differed in their abundance and growth dynamics with respect to land-use. Meadow soil supported more bacterial growth, higher growth efficiency, and greater overall mortality than agricultural soils, pointing toward more efficient conversion of SOM into microbial necromass which contributes to long-term C stabilization.

## Supplementary Material

Wattenburger2024_SI_ycaf014

## Data Availability

The DNA amplicon sequence dataset generated and analyzed during the current study is available in the NCBI Sequence Read Archive (Accession: PRJNA910174) [https://www.ncbi.nlm.nih.gov/bioproject/PRJNA910174]. Other datasets generated or used in this study are available from the corresponding author on reasonable request.
