# Supplementary material for: Dynamics of bacterial growth, and life-history tradeoffs, explain differences in soil carbon cycling due to land-use
Source: ISME Commun. 2025 Jan 30;5(1):ycaf014. doi: 10.1093/ismeco/ycaf014 (PMC11844245; doi:10.1093/ismeco/ycaf014)
Supplement: Wattenburger2024_SI_ycaf014 [file wattenburger2024_si_ycaf014.pdf]

**Dynamics of bacterial growth, and life-history tradeoffs, explain differences in soil carbon cycling due to land-use**

Cassandra Wattenburger, Evangeline Wang, Daniel H. Buckley

## Supplementary Information

**Table S1.** Parameter estimate definitions and average values measured in each soil.

| **Parameter estimate** | **Definition and units** | **Soil system** | **Avg ± sd** | **Range** |
|---|---|---|---|---|
| Generation time (*g*) | Days needed to double population size | Agriculture | 7.13 ± 0.92 | 6.38, 8.16 |
| | | Meadow | 6.88 ± 0.51 | 6.34, 7.34 |
| Lag time | Days from litter addition until the start of growth | Agriculture | 3.85 ± 0.52 | 3.31, 4.36 |
| | | Meadow | 4.51 ± 0.70 | 3.76, 5.14 |
| $\Delta N_g$ | Change in normalized abundance due to growth | Agriculture | 0.020 ± 0.002 | 0.018, 0.021 |
| | | Meadow | 0.036 ± 0.003 | 0.032, 0.038 |
| Halving time (*h*) | Days needed to halve population size | Agriculture | 9.14 ± 1.33 | 8.04, 10.63 |
| | | Meadow | 8.00 ± 1.20 | 7.11, 9.37 |
| Start of death | Days from litter addition until the start of death | Agriculture | 5.78 ± 0.74 | 4.94, 6.30 |
| | | Meadow | 5.78 ± 0.78 | 4.86, 6.27 |
| $\Delta N_d$ | Change in normalized abundance due to death | Agriculture | 0.016 ± 0.002 | 0.014, 0.018 |
| | | Meadow | 0.030 ± 0.004 | 0.024, 0.031 |

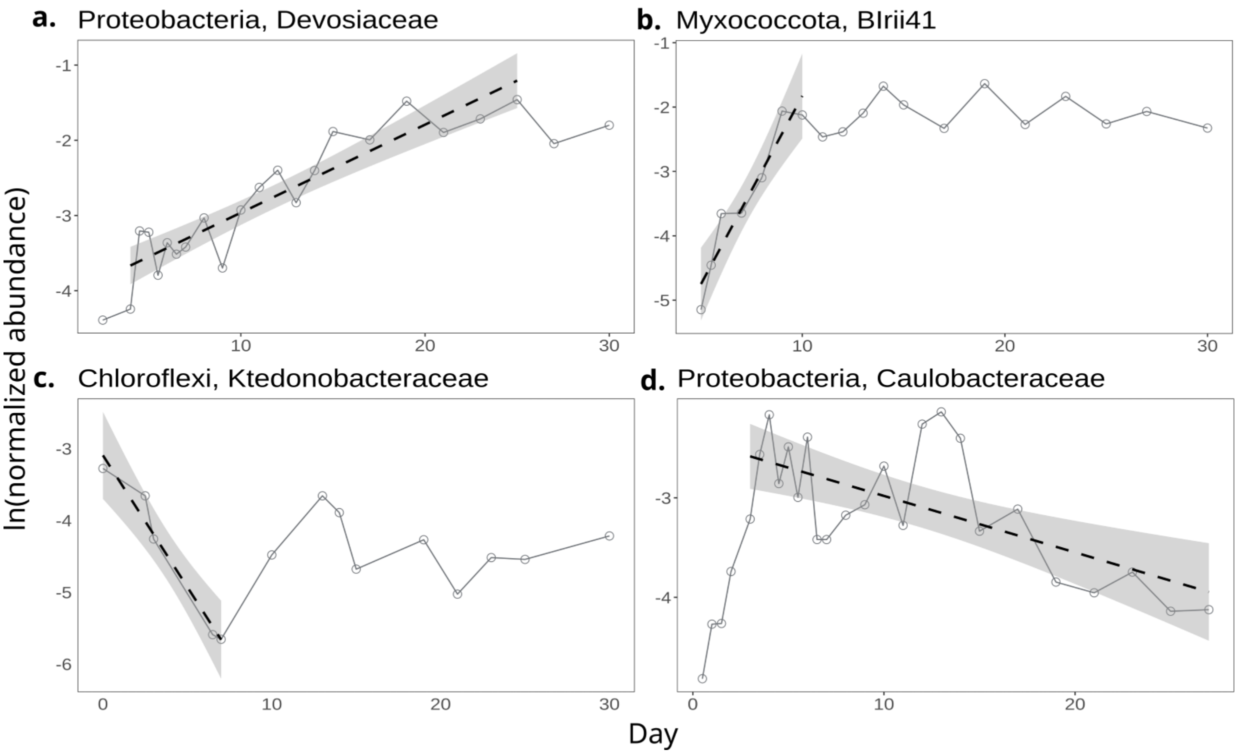


**Figure S1.** Examples of estimated growth (**a, b**) and death (**c, d**) for individual ASVs. Each time series represents the normalized abundances of an individual ASV in a replicate over time. Dashed lines indicate growth inference (as described in methods) from which we can estimate rate of growth (or death), start time for growth (or death), end time for growth (or death), and the abundance of the population when growth starts or ends. Plots are labelled with phylum and family membership of the ASV.

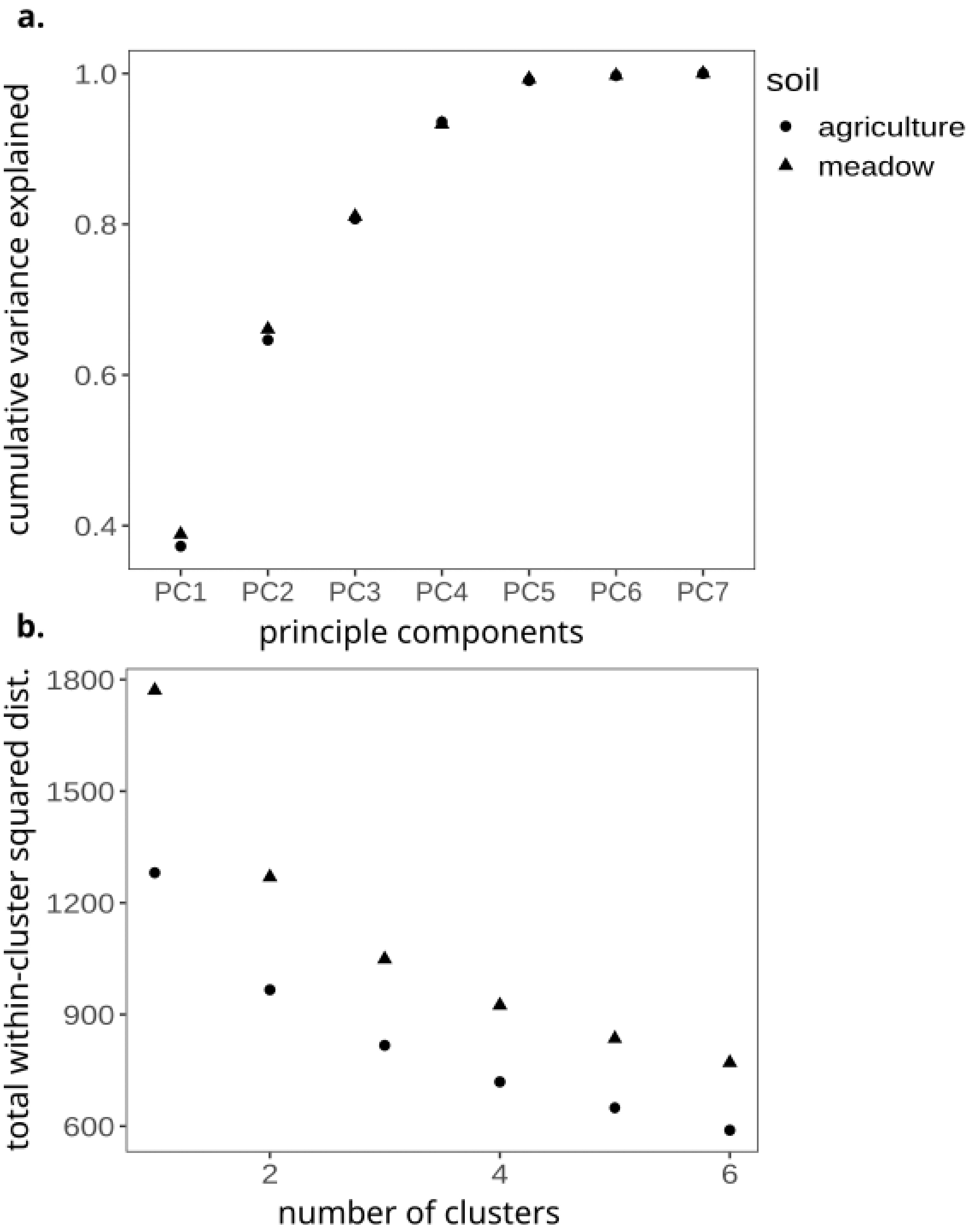


**Figure S2.** Three clusters were chosen as most representative for the life history groupings based on the elbow method using **(a)** principal components analysis prior to clustering and **(b)** the total within-cluster squared distance at various cluster numbers.

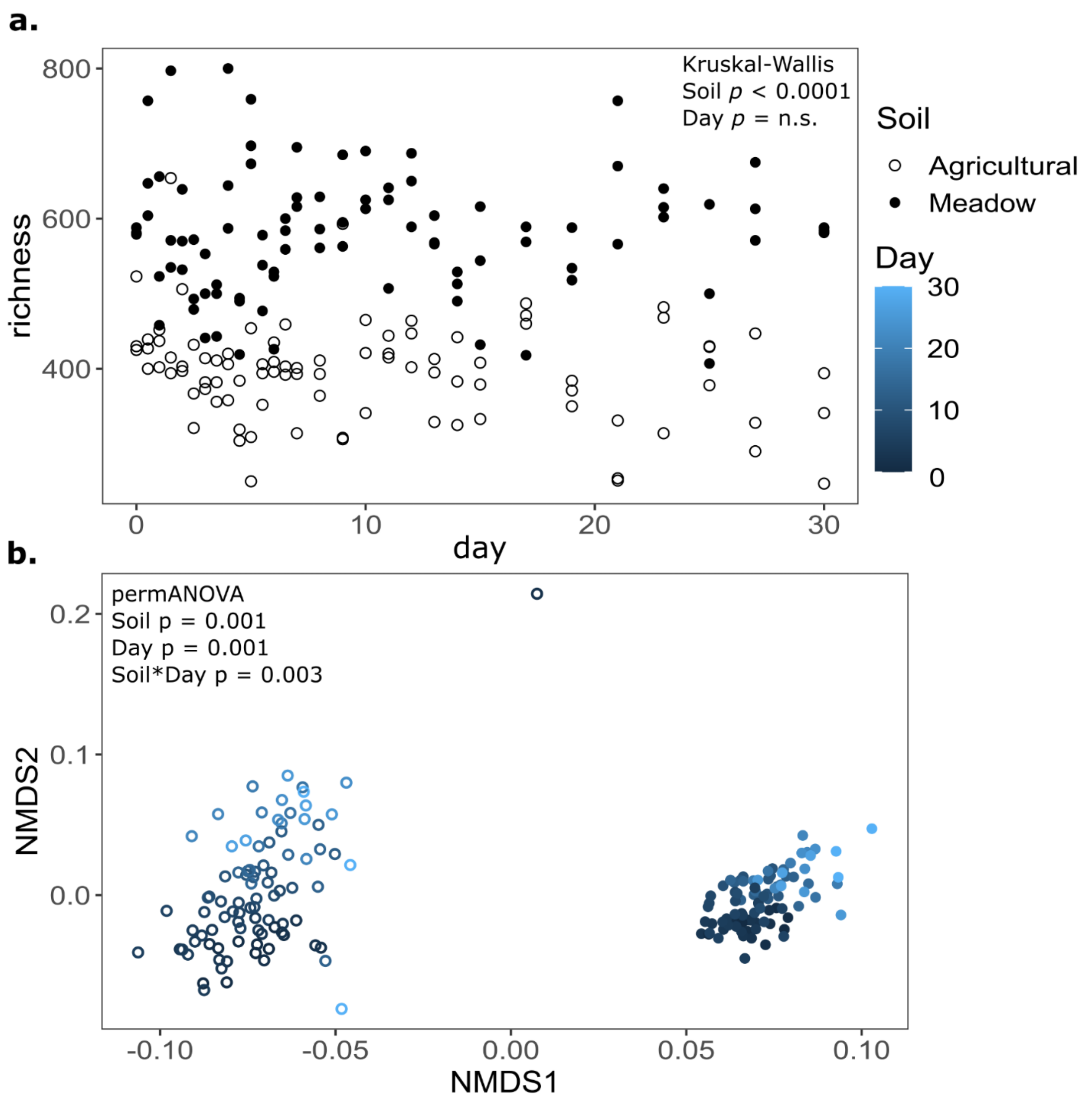


**Figure S3.** Richness (measured as taxa observed) of informative ASVs (**a**) was higher in the meadow soil than in the agricultural soil, and community composition as assessed by weighted unifrac distance (**b**) was significantly affected by soil, day, and their interaction. Symbol fill is defined in the legend.

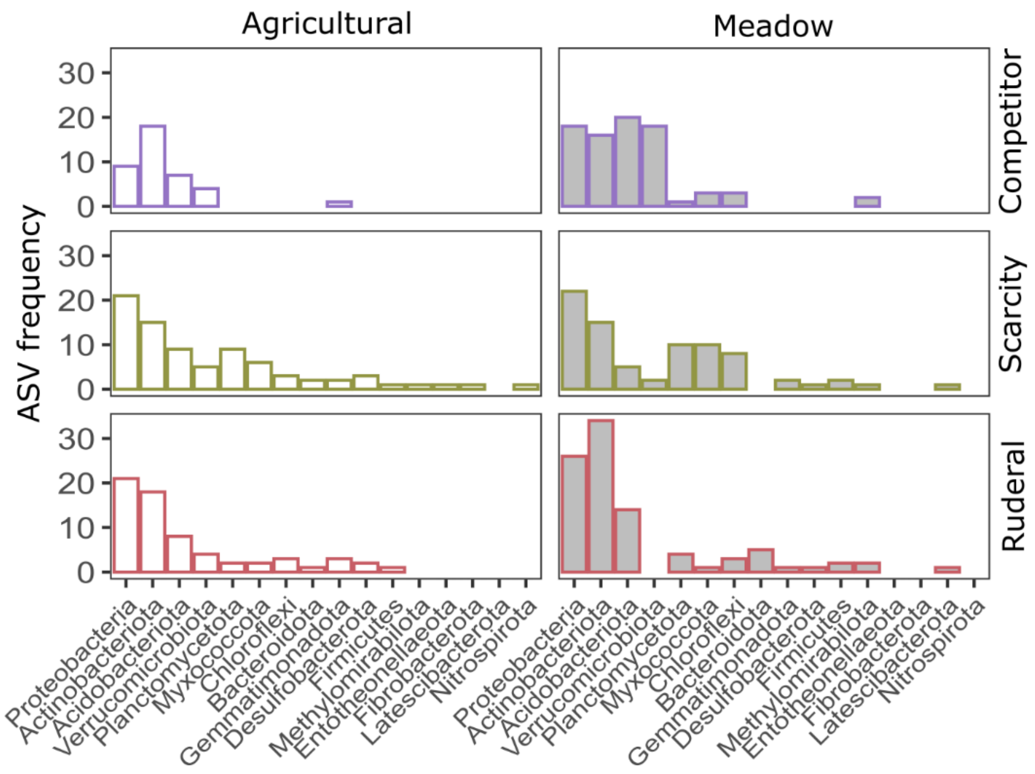


**Figure S4.** Phylogenetic membership of growth-estimated ASVs in the agricultural and meadow soils. Cluster membership was defined consistent with **Figure 2**, as described in methods.

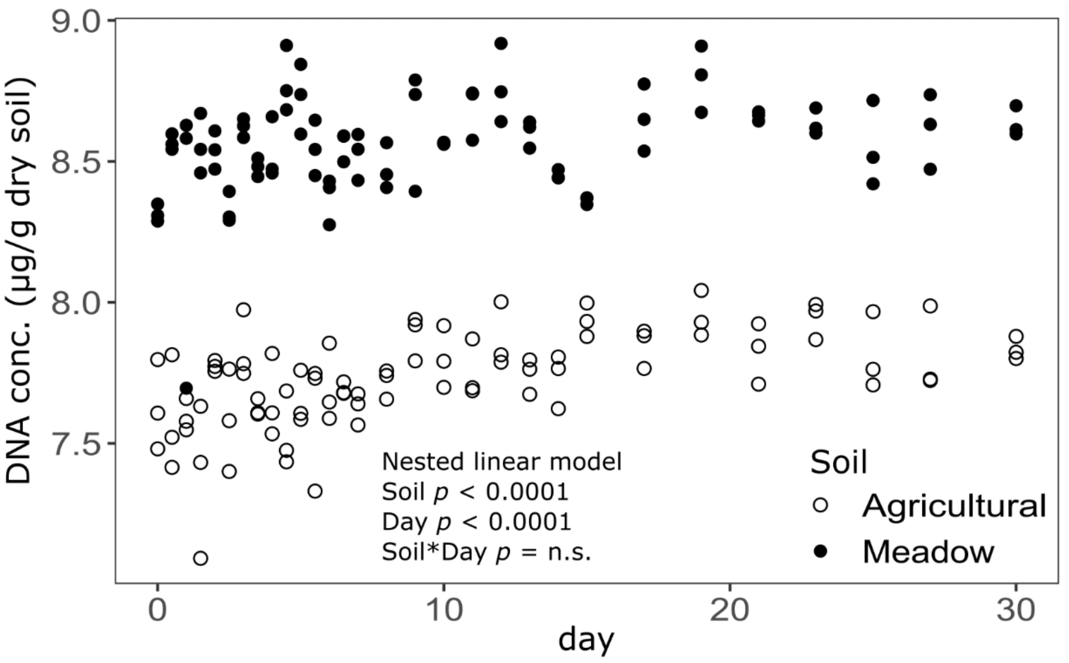


**Figure S5.** The meadow soil yielded higher DNA concentrations than the agricultural soil, and soil DNA yield increased in microcosms over time. Each point represents the DNA yield from an individual microcosm.

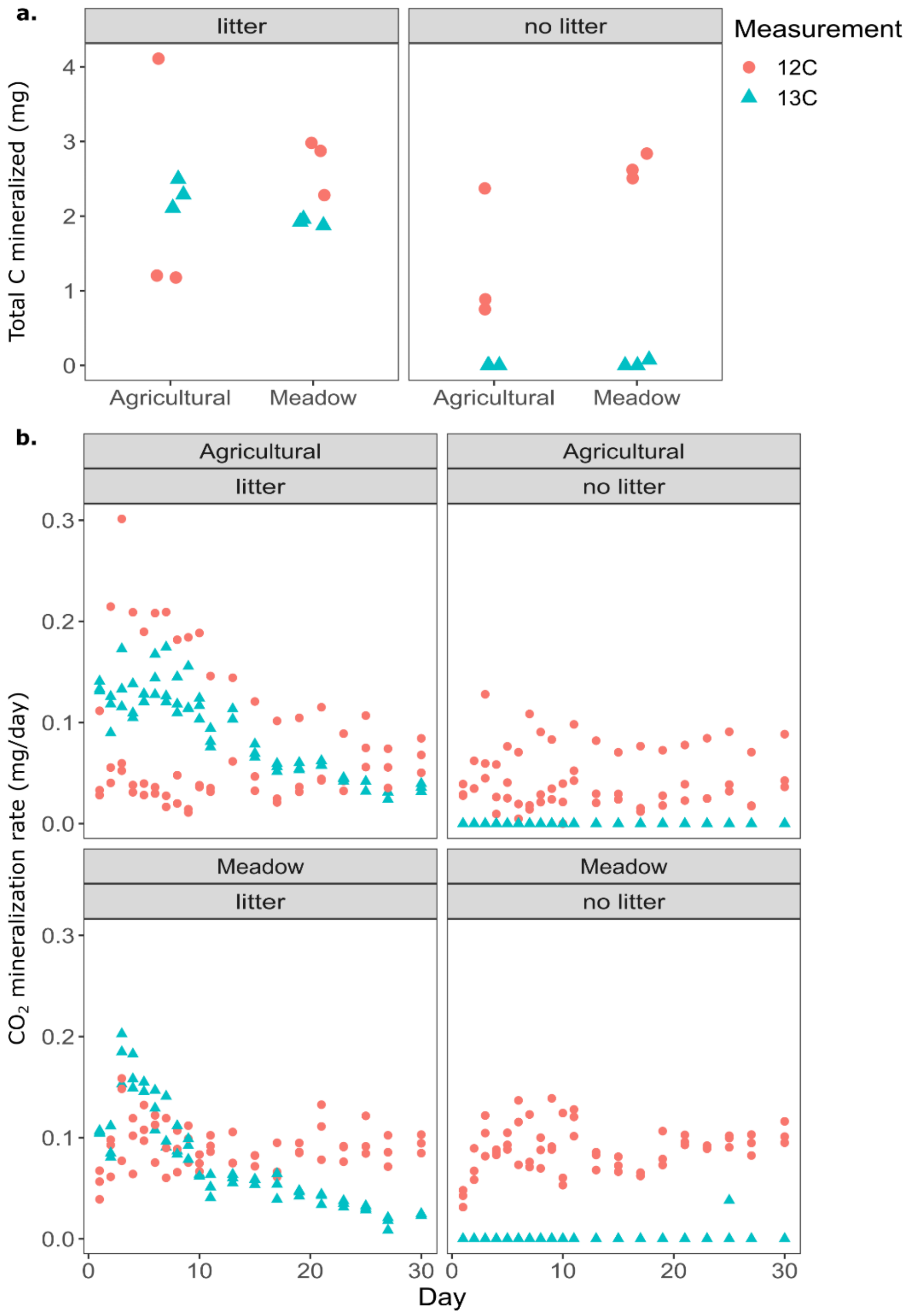


**Figure S6.** Total C mineralized as $CO_2$ (**a**) and $CO_2$ mineralization rate (**b**) from agricultural and meadow soils. $^{13}C$-litter was used as described in methods. No significant differences between soils, as tested by Welch *t*-tests, were detected for either $^{13}CO_2$ or $^{12}CO_2$ owing to high variation among replicates from the agricultural soil.
